# Cross-Bioaugmentation Among Four Remote Soil Samples Contaminated With Oil Exerted Just Inconsistent Effects on Oil-Bioremediation

**DOI:** 10.3389/fmicb.2019.02827

**Published:** 2019-12-05

**Authors:** Dina M. Al-Mailem, Mayada K. Kansour, Samir S. Radwan

**Affiliations:** Microbiology Program, Department of Biological Sciences, Faculty of Science, Kuwait University, Kuwait City, Kuwait

**Keywords:** oil-bioremediation, cross-bioaugmentation, diazotrophs, hydrocarbonoclastic bacteria, remote soils

## Abstract

Soil samples were collected from Kuwait, Lebanon, Egypt, and Germany, and artificially polluted with 3% (w/w) crude oil. Cross-bioaugmentation was done among them, and the oil-consumption and the constituent communities of hydrocarbonoclastic bacteria were monitored periodically through 6 months. The results showed that cross-bioaugmentation did not bring about reproducible effects on oil-removal in the four soils. After 6 months, oil-removal values reached between 82 and 95% in most of the samples including the unbioaugmented controls. The numbers of hydrocarbonoclastic bacteria showed significant increases followed by significant decreases during the course of bioremediation also in the unbioaugmented controls. In most cases, the inoculated bacterial taxa failed to colonize the soils, and oil-removal was achieved mainly by the native (autochthonous) soil bacterial communities. those belonged to the genera *Mycolicibacterium*, *Mycobacterium*, *Xanthobacter*, *Pseudoxanthomonas*, *Pseudomonas*, *Zavarzinia*, and others. The microbial communities in the four soils also comprised nitrogen fixing bacteria belonging to the genera *Gordonia*, *Rhizobium*, *Kocuria*, and *Azospirillum*. Such diazotrophs are known to enrich the soils with fixed nitrogen and thus, contribute to enhancing the microbiological hydrocarbon-consumption. It was concluded that cross-bioaugmentation leads to unpredictable and inconsistent effects on oil removal. Therefore, it could not beregarded as the technology of choice for oil-bioremediation.

## Introduction

There are physical and mechanical means currently available for removal of crude oil polluting the environment. Those include the use of skimmers, oil-booms, sorbents and dispersants, in addition to incineration, land filling and others (Wardley-Smith, 1983; Al-Majed et al., 2012; Prendergast and Gschwend, 2014). These approaches are, however, not cost-effective (Rosenberg, 1993), and many of them may lead to additional environmental pollution. Thus, oil-dispersants may be inflammable, toxic and may lead to fouling of coastal areas (National Research Council [NRC], 1989; Cordes et al., 2016). Incineration causes air-pollution and land filling may contribute to ground-water pollution with leachates (Ugwoha and Omenogor, 2017). On the other hand, bioremediation is commonly considered as a much more cost-effective and environmentally safe approach for the removal of spilled oil.

Bioremediation makes use of the activities of microorganisms in the mineralization of organic pollutants (Atlas, 1995; Atlas and Bartha, 1998; Khan et al., 2013; Al-Wasify and Hamed, 2014). Oil-spills lead automatically to enriching the polluted sites with native (autochthonous) hydrocarbonoclastic microorganisms, which is reflected in enhanced oil-removal especially when essential microbial requirements (moisture, nutrients, etc.) are satisfied. However, many researchers in the field of oil-bioremediation believe that inoculating oily sites exogenously with any hydrocarbon degrading microorganisms, an approach known as bioaugmentation, should enhance the oil-removal (Van et al., 1998; Kapley and Purohit, 2001; Moharikar et al., 2003; Domde et al., 2007; Szulc et al., 2014; Ugwoha and Omenogor, 2017). Therefore, such researchers use either pure single cultures, consortia of pure cultures especially of *Pseudomonas* spp. or mixtures of unidentified microorganisms (Van et al., 1998; Khan et al., 2013; Ugwoha and Omenogor, 2017). Culture cocktails are available commercially (Applied Biotreatment Association, 1989, 1990). The organisms must of course be capable of colonizing, survival and propagation in the inoculated site, which is, however, not always the case. Ecological microbiologists are aware of the severe competition facing exogenous microorganisms by the native (autochthonous) inhabitants. Rather recently, the strategy of autochthonous bioaugmentation (ABA) has been suggested for oil-bioremediation (Hosokawa et al., 2009). As the term indicates, only autochthonous (indigenous to the polluted environment) microorganisms are capable of colonizing and inhabiting oily sites successfully and are thus, capable of removing oil (Hosokawa et al., 2009; Nikopoulou et al., 2013; Di Gregorio et al., 2014). The ABA-strategy is obviously based on the ecological classification of soil microorganisms in autochthonous and allochthonous inhabitants (Winogradsky, 1924). In contrast to the latter, the autochthonous microorganisms are well adapted to the conditions in the concerned environment and thus, contribute effectively to biochemical activities therein.

Through about three decades of research on the bioremediation of marine and desert areas suffering from the greatest man-made oil-spill which was one of the consequences of the second Gulf War (August 1990–February 1991), our group in Kuwait collected research experience in this field (for reviews see: Radwan and Sorkhoh, 1993; Radwan and Al-Hasan, 2000; Radwan, 2008, 2009; Al-Mailem D. et al., 2015; Al-Mailem and Radwan, 2016). Two major conclusions, which are actually two faces of one coin, were made. Firstly, the best bioremediation results are obtained when this biotechnology depends on the activities of native hydrocarbonoclastic microbial communities. Secondly, inoculation with exogenous microorganisms leads to unpredictable, in many cases also negative effects on oil-removal.

The major objective of this work is to shed new light on the feasibility of bioaugmentation for removal of crude oil spilled in soil, especially in view of the fact that the spilled oil automatically enriched the soil with autochthonous hydrocarbonoclastic inhabitants as mentioned above. For this, we collected four soil samples from three different continents, polluted them artificially with crude oil and studied in bench-scale batch-cultures their bioremediation via mutual cross-bioaugmentation. This study was proposed to contribute to answering the question: could soil microorganisms from remote areas (i.e., adapted since ages to specific physicochemical parameters) colonize and remove oil in another foreign soil? This question seemed important to answer given the repeated claims in the old and recent literature about significant enhancement of oil-removal in polluted environments after their inoculation with exogenous oil-degrading microorganisms. Surprisingly, there are currently bacterial cocktails available commercially for oil-bioremediation purposes, as already mentioned.

## Materials and Methods

### The Studied Soil Samples

Pristine soil samples were collected in sterile containers from Kuwait-Asia (a desert soil sample from Al-Ahmadi area, 33 km south of Kuwait City), Lebanon-Asia (a garden soil sample from Al-Janoub area, 120 km south of Beirut), Egypt-Africa (a garden soil sample from a village, 120 km north of Cairo), and Germany-Europe (a sunflower-field soil sample at Münster/Westf, 850 km west-south of Berlin). Physicochemical parameters were measured using TOC-VCPH, Shimadzu for total organic carbon, CHNS-Vario Micro Cube for total nitrogen, Lachat quickchem-8500 Series II for total phosphorus, ASS, Perkin Elmer model: Pinnacle 900F for metal, ICP-OES-7300DV for As and Hg Analyzer Hydra-II, Leeman for Hg analysis.

### Soil Samples for Bioaugmentation

To prepare inocula for cross-bioaugmentation each of, 100 g aliquot of the four remote soils was mixed with 3% crude oil and 100 ml sterile tap water. The sealed flasks were incubated at room temperature (about 27°C) for 7 months on an electrical shaker, 120 rpm to enrich them with native hydrocarbonoclastic microorganisms. The content of each flask was further homogenized by vigorous, manual agitation and 1 ml portions of those common inocula were used for cross-bioaugmentation purposes. During the 7 months on the electrical shaker, biosurfactants were produced which also contributed to the inoculum homogeneity.

### Batch-Culture Setup for Bioremediation

For bench-scale bioremediation, 100 g aliquots of the pristine soils were suspended in 100 portions of sterile water in 250 ml conical flasks and mixed with 3 g portions of light Kuwaiti crude oil (Natural Oil Company of Kuwait). Each flask was inoculated with 1 ml of one of the common inocula from the other three soil samples. The setup also included unbioaugmented flasks and flasks with autoclaved inocula (abiotic) as controls. Three replicates were prepared throughout. The flasks were Teflon sealed to avoid oil-volatilization and were incubated on an electrical shaker, 120 rpm, under room conditions (about 27°C). At time zero, and after 3 and 6 months, triplicate flasks were harvested for measurement of oil-consumption and microbiological analysis.

### Measurement of Oil-Consumption

The residual oil in the contents of each flask was recovered by extraction with three successive portions of 15 ml pentane. The volume of the combined extract was raised to 50 ml with pure pentane and 1 μl was analyzed by gas liquid chromatography (GLC). Hydrocarbon-consumption was expressed in terms of percentage of total peak-area reduction in the samples based on the peak areas of the controls (time-zero flasks and abiotic controls in which the bacteria were killed by autoclaving). The GLC was done using a Chrompack (NJ, United States) CP-9000 instrument equipped with a FID, a WCOT fused silica CP-Sil capillary column, and a temperature program of 45–310°C, raising the temperature at a rate of 10°C min^–1^.

### Analysis of Hydrocarbonoclastic Communities

For counting hydrocarbonoclastic bacteria, the dilution-plating method on a solid mineral medium with oil vapor as a sole carbon and energy source was used (Sorkhoh et al., 1990). A dilution series was prepared from the content of each culture. Aliquots, 0.1 ml of each dilution, were spread on the surface of the agar mineral medium in Petri-dishes, and oil vapor was made available as a sole organic substrate from 3 ml oil-impregnated filter papers fixed in the dish lids. Dishes were sealed with cello-tape to avoid oil loss, inverted and incubated at 30°C for 12 days. Five parallel plates were prepared for each dilution. The colony forming units (CFU) were counted. Strains in the pooled replicate plates were differentiated according to their colony and cell morphologies and their staining reaction characteristics. They were counted and their percent in the total count calculated. Representative colonies were isolated, purified and maintained on the mineral medium containing 1% crude oil. The isolates were subcultured every 15 days.

For characterization of the isolates, their 16S rRNA-genes were sequenced and the sequences compared with those of type strains in the GenBank data base following established procedure (Altschul et al., 1997; Santegoeds et al., 1998; Al-Mailem D.M. et al., 2015). Details of the procedure and the accession numbers under which the sequences were deposited in the GenBank are available in the Supplementary Material.

### Statistical Analysis

Triplicate determinations for each analysis were done and the mean values, ± standard deviation values, were calculated using Microsoft Excel 2007. Statistical Package for Social Sciences, version 12, was used to assess the degree of significance. The analysis of variance (ANOVA) was used to differentiate between the means of the tested parameters.

## Results and Discussion

### The Studied Soil Samples

Table 1 shows how different the physicochemical parameters of the four soil samples were. With the exception of the heavy metal concentration values, all other parameters showed comparatively large variations. In view of the big differences of the moisture contents of the studied soils, and for the purpose of proper comparison, we recalculated all our analytical data on dry soil bases.

**TABLE 1:** Some physicochemical parameters of the studied pristine soils.

**Parameter**	**Kuwaiti soil**	**Lebanese soil**	**Egyptian soil**	**German soil**
Soil type	Sandy	Loamy	Loamy	Loamy
Temperature (°C)^∗^	39	28	33	21
pH	6.5	7.1	8.2	7.5
Moisture (%)	10.5	5.1	13.3	41.7
Total organic carbon (%)	0.9	1.1	1.3	13.1
Total extractable	0.3	<0.1	0.1	<0.1
hydrocarbons (%)^∗∗^				
Total nitrogen (%)	0.1	0.2	0.3	1.5
Ca (g kg^–1^)	26.7	7.4	5.9	19.4
Mg (g kg^–1^)	7.0	2.6	13.9	6.2
K (g kg^–1^)	0.5	2.0	5.2	0.7
P (g kg^–1^)	0.3	0.8	1.3	0.9
Cd (mg kg^–1^)	2.0	3.4	3.1	1.7
As (mg kg^–1^)	<0.1	<0.1	<0.1	<0.1
Pb (mg kg^–1^)	<1	<1	<1	<1
Hg (μg kg^–1^)	<0.2	<0.2	0.4	<0.2

*^∗^Maximum outdoor temperature in the last week of August, the time of sampling; ^∗∗^with pentane.*

### Batch-Culture Bioremediation

Table 2 contains the results of oil-consumption in the unbioaugmented (control) and bioaugmented soil samples under investigation. In the middle of the bioremediation period, i.e., after 3 months, the proportions of the removed oil in the unbioaugmented batches ranged between 22 (Egyptian soil) and 53 (German soil)%. Depending on the bioaugmentation material used, the proportions of removed oil decreased or increased significantly (ANOVA, *n* = 3, *P* < 0.05) in each of the four samples. Thus, cross-bioaugmentation of the Kuwaiti soil with the German soil inhibited the process of bioremediation, but bioaugmentation of this soil with the Lebanese and Egyptian soils enhanced it. Oil-removal in the Lebanese soil was significantly inhibited by cross-bioaugmentation with the Kuwaiti, Egyptian and German soils (ANOVA, *n* = 3, *P* < 0.05). In the Egyptian and German soils, oil-removal was significantly enhanced by all of the cross-bioaugmentation treatments (ANOVA, *n* = 3, *P* < 0.05). This trend, however, did not continue till the end of the bioremediation period. After 6 months, the highest oil-removal result in the Kuwaiti soil was in the unbioaugmented control, and the lowest in the German soil bioaugmented batch. Within this context, we found in a preliminary experiment that bioaugmentation of oily desert soil from Kuwait with a similar soil with long history of oil pollution (i.e., enrichment with autochthonous hydrocarbonoclastic bacteria did not bring about any significant increase of oil-removal). A relatively limited enhancement of oil-removal occurred in the Lebanese soil samples bioaugmented with the Kuwaiti, Egyptian, and German soils. Cross-bioaugmentation also brought about enhancement in oil-removal in the Egyptian soil. Inoculation of the German soil with the Kuwaiti and Lebanese soils brought about a limited enhancement of oil-bioremediation, but with the Egyptian soil a significant inhibition (ANOVA, *n* = 3, *P* < 0.05).

**TABLE 2:** Oil consumption in the four remote soil samples during bioremediation via cross-bioaugmentation.

**Soil sample**	**Bioaugmented**	**Percentage of consumed**	
**from**	**with**	**oil (%) after**	
		**3 months**	**6 months**
Kuwait	(Unbioaugmented)	32.2 ± 0.3	91.1 ± 1.2
	Lebanese soil	81.3 ± 0.5	81.8 ± 0.6
	Egyptian soil	83.0 ± 0.9	83.2 ± 0.8
	German soil	15.7 ± 0.4	71.4 ± 0.4
Lebanon	(Unbioaugmented)	27.1 ± 1.1	48.2 ± 1.5
	Kuwaiti soil	10.6 ± 0.1	63.8 ± 0.9
	Egyptian soil	1.5 ± 0.7	59.1 ± 0.2
	German soil	2.4 ± 0.3	60.6 ± 0.2
Egypt	(Unbioaugmented)	22.4 ± 0.3	69.4 ± 0.5
	Kuwaiti soil	30.9 ± 0.9	94.0 ± 0.6
	Lebanese soil	57.6 ± 1.0	93.1 ± 1.3
	German soil	54.6 ± 0.2	93.1 ± 0.7
Germany	(Unbioaugmented)	52.6 ± 0.5	86.0 ± 0.1
	Kuwaiti soil	75.3 ± 0.5	94.6 ± 0.9
	Lebanese soil	77.8 ± 0.3	91.1 ± 1.4
	Egyptian soil	67.3 ± 1.2	76.2 ± 0.7

*Data are means of three replicates ± standard deviation values.*

A question arises regarding the inconsistency of the oil-removal values according to the type of soil. One factor could be the differences in the physicochemical parameters in the four studied soils, as represented by the few ones we measured in this study. Those differences affect the microbial activities. To mention but a few examples, it is well documented that nitrogen and phosphorus bio-enhance the microbial hydrocarbon-biodegradation (Radwan, 2008, 2009; Al-Awadhi et al., 2012). It has also been reported that minerals viz Ca^2+^ (Al-Mailem D.M. et al., 2015), Mg^2+^ and K^+^ (Perfumo et al., 2007) under certain conditions may enhance hydrocarbon-mineralization by bacteria. Conventional organic matter may inhibit hydrocarbon removal when utilized by the organisms as alternative substrates. Meanwhile, organic compounds may promote oil-removal by amplifying the native (autochthonous) hydrocarbonoclastic communities which prefer them as substrates (Radwan et al., 2019). Another probable factor could be the interaction of the native with the exogenous microbial communities. This interaction may be expressed as competition with inhibitory effects or as cooperation with promotive effects on the oil biodegradation. The main conclusion from the latter results is that cross-bioaugmentation is associated with inconsistency of the bioremediation results. This unpredictability is certainly a limitation of bioaugmentation as an oil-bioremediation approach.

### Numbers of Hydrocarbonoclastic Bacteria During Bioremediation

**FIGURE 1:**
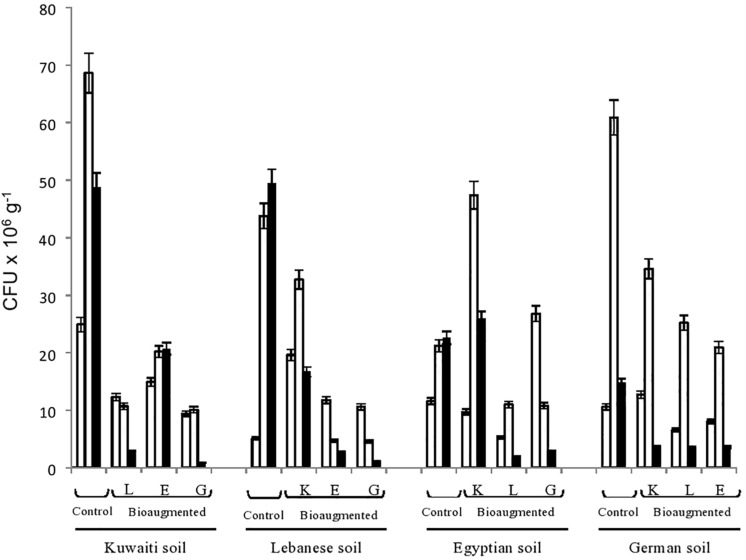
Numbers of hydrocarbonoclastic bacteria in the four remote, oily soil samples during their bioremediation via cross-bioaugmentation. Open columns, time zero; dotted columns, 3 months; closed columns, 6 months of bioaugmentation. L, Lebanese; E, Egyptian; K, Kuwaiti; G, German soils for cross-bioaugmentation.

Figure 1 presents the numbers of hydrocarbon-utilizing bacteria in the four studied soils as affected by cross-bioaugmentation. To recall, those numbers were counted on a selective mineral medium with oil vapor as a sole organic substrate. In other words, all isolates were hydrocarbonoclastic. This was confirmed by the fact that no colonies at all showed up in the inoculated control dishes which were deprived of oil-vapor.

The CFU in all the batches were rather numerous, ranging in number between a few millions to more than 60 million g^–1^ soil. In most cases, the highest numbers were counted at the end of the third month. The CFU numbers then tended to decrease toward the end of the experiment, probably due to nutrient- and oxygen-depletion and toxic metabolite accumulation, but they still remained in the range of millions g^–1^. These dynamic changes in the numbers, whose details are clear in Figure 1 confirm the involvement of the various bacterial communities in oil-biodegradation. The increases and decreases in numbers were statistically significant (ANOVA, *n* = 5, *P* < 0.05).

### Dynamics of Bacterial Communities During Bioremediation of the Kuwaiti Soil

As mentioned above, bioaugmentation means the inoculation of exogenous microorganisms into the polluted site (Szulc et al., 2014) resulting in the addition of more gene pools therein (Domde et al., 2007). After consulting the literature, it was clear that cross-bioaugmentation among remote areas has not been done before the way we did in this study. Although soil samples already enriched with hydrocarbonoclastic bacteria were used for inoculation, no oil-removal enhancement occurred in most of the cases. This was obviously due to the failure of the exogenous bacteria to withstand the competition with the already existing microorganisms (Radwan, 1990). An earlier study in our laboratory showed that inoculated *Arthrobacter* strains imported from Germany failed to colonize oily Kuwaiti soil, while locally isolated *Arthrobacter* strains did (Radwan et al., 1997). This failure seemed to be the rule in the current study, in which the dynamics of bacteria during bioremediation was studied.

**FIGURE 2:**
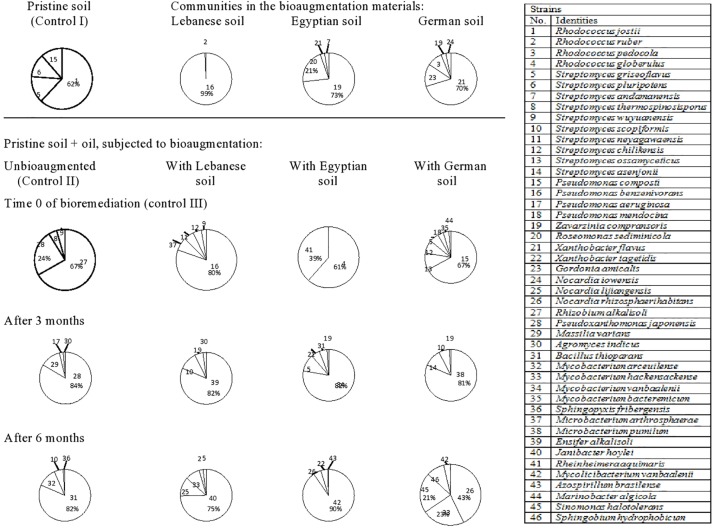
Dynamics of hydrocarbonoclastic bacterial communities in the oil contaminated soil sample from Kuwait during bioremediation via cross-bioaugmentation.

Totally, 46 species of hydrocarbonoclastic bacteria were isolated from the unbioaugmented and bioaugmented Kuwaiti soil samples (Figure 2, refer also to the Supplementary Material). They belonged mainly to the following genera arranged in decreasing order of species numbers: 10 *Streptomyces*, four each of *Rhodococcus*, *Mycobacterium*, and *Pseudomonas*, three *Nocardia*, and two *Xanthobacter* spp. Other genera were presented by one species, each. The pristine Kuwaiti soil harbored mainly *Rhodococcus jostii* (strain 1), *Streptomyces pluripotens* (strain 6), *Streptomyces griseoflavus* (strain 5), and *Pseudomonas composti* (strain 15). The mere addition of oil into this soil resulted in a completely different composition. The predominant taxa were *Rhizobium alkalisoli* (strain 27), *Pseudoxanthomonas japonensis* (strain 28), *Streptomyces thermospinosisporus* (strain 8), and *Streptomyces wuyuanensis* (strain 9). Being instantaneous (at time zero), this change could have not resulted from the propagation of the latter four species it was probable physical. The oil added created hydrophobic conditions that released those four organisms from less hydrophobic soil cores. It is well documented that many hydrocarbonoclastic bacteria have hydrophobic cell envelopes (Van Loosdrecht et al., 1987; Castellanos et al., 1997). After 3 months of bioremediation, *P. japonensis* (strain 28) and after 6 months, *Bacillus thioparans* (strain 31) took over the absolute predominance.

The Kuwaiti soil bioaugmented with the Lebanese soil sample harbored mainly *Pseudomonas benzenivorans* (strain 16) which succeeded in colonizing the Kuwaiti soil at time 0, but it disappeared afterward. Probably it could not stand the competition with the native Kuwaiti soil species. After 3 months of bioremediation, *Ensifer alkalisoli* (strain 39) and after 6 months, *Janibacter hoylei* (strain 40) took over the absolute predominance. Hence, they may be expected to be major oil biodegraders in the Kuwaiti soil.

The Kuwaiti soil bioaugmented with the Egyptian soil harbored two predominant taxa, *Zavarzinia compransoris* (strain 19) and *Roseomonas sediminicola* (strain 20) and two minor taxa, *Xanthobacter flavus* (strain 21), and *Streptomyces andamanensis* (strain 7). With the exception of *X. flavus* (strain 21); none of those bioaugmented taxa could colonize or persist in the Kuwaiti soil. After 3 months of bioremediation, *Mycobacterium vanbaalenii* (strain 34) and after 6 months, *Mycolicibacterium vanbaalenii* (strain 42) took over the absolute predominance.

The Kuwaiti soil bioaugmented with the German soil harbored as predominant species *X. flavus* (strain 21) and as less dominant ones, *Gordonia amicalis* (strain 23), *Rhodococcus pedocola* (strain 3), *Z. compransoris* (strain 19), and *Nocardia iowensis* (strain 24). Also those taxa, failed to colonize or persist in the Kuwaiti soil. After 3 months of bioremediation, *Microbacterium pumilum* (strain 38) and after 6 months, *Nocardia rhizosphaerihabitans* (strain 26), *Mycobacterium hackensackense* (strain 33), and *Sinomonas halotolerans* (strain 45) became predominant.

### Dynamics of Bacterial Communities During Bioremediation of the Lebanese Soil

**FIGURE 3:**
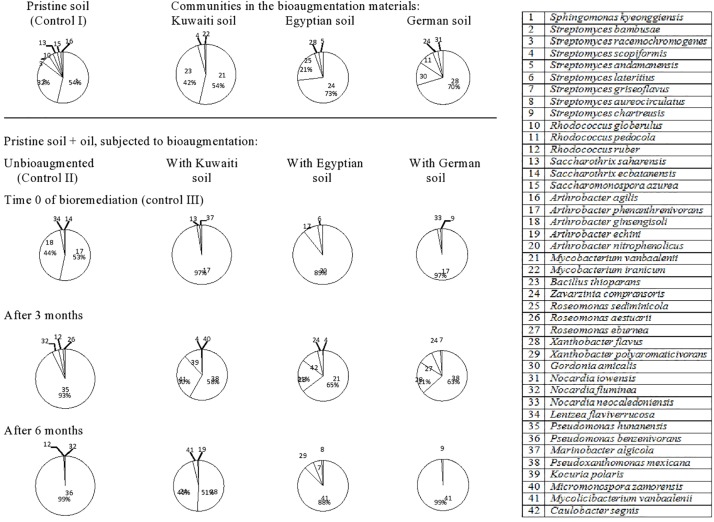
Dynamics of hydrocarbonoclastic bacterial communities in the oil contaminated soil sample from Lebanon during bioremediation via cross-bioaugmentation.

The 42 hydrocarbon degrading bacterial species isolated from the Lebanese soil (Figure 3, refer also to Supplementary Material) belonged to the following genera: *Streptomyces* (eight species) > *Arthrobacter* (five species) > *Rhodococcus* (three species), *Roseomonas* (three species), *Nocardia* (three species) > *Saccharothrix* (two species), *Mycobacterium* (two species), *Xanthobacter* (two species), and *Pseudomonas* (two species). Other genera were represented with one species, each. The pristine soil (control) harbored *Sphingomonas kyeonggiensis* (strain 1) and *Streptomyces bambusae* (strain 2) as the predominant taxa. The mere addition of oil to the soil probably released from absorption on soil cores the two taxa, *Arthrobacter phenanthrenivorans* (strain 17) and *Arthrobacter ginsengisoli* (strain 18) making them absolutely predominant at time zero. However, none of the four species was detected in the soil and the predominance was taken over by *Pseudomonas hunanensis* (strain 35) after 3 months and *P. benzenivorans* (strain 36) after 6 months.

The Lebanese soil inoculated with the Kuwaiti soil harbored *M. vanbaalenii* (strain 21) and *B. thioparans* (strain 23) as dominant species, but *A. phenanthrenivorans* (strain 17) took over the predominance in the oily soil at time zero. None of the three taxa was detected in that soil at later phases and the predominance was taken over by *Pseudoxanthomonas mexicana* (strain 38), *M. vanbaalenii* (strain 41) and *Kocuria polaris* (strain 39) after 3 months, and by *X. flavus* (strain 28) and *Z. compransoris* (strain 24) after 6 months.

The Lebanese soil bioaugmented with the Egyptian soil contained *Z. compransoris* (strain 24) and *R. sediminicola* (strain 25) as predominant hydrocarbonoclastic species. However, none of both taxa showed up in the oily soil at time zero, and instead, *Arthrobacter nitrophenolicus* (strain 20) and *A. phenanthrenivorans* (strain 17) predominated. Also here, other species, viz *M. vanbaalenii* (strain 21), *X. flavus* (strain 28), and *Caulobacter segnis* (strain 42) predominated after 3 months and *M. vanbaalenii* (strain 41) after 6 months.

The Lebanese soil inoculated with the German soil contained as predominant bacteria *X. flavus* (strain 28), *G. amicalis* (strain 30), and *R. pedocola* (strain 11). In the oily soil at time zero, *A. phenanthrenivorans* (strain 17) predominated. However, in the third month, *P. mexicana* (strain 38), *X. flavus* (strain 28), and *Roseomonas eburnean* (strain 27) took over the predominance and in the sixth month, *M. vanbaalenii* (strain 41) was absolutely dominant.

### Dynamics of Bacterial Communities During Bioremediation of the Egyptian Soil

**FIGURE 4:**
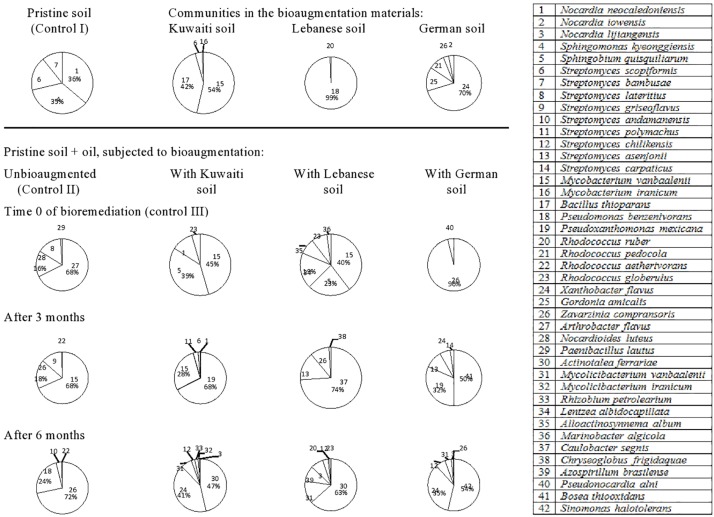
Dynamics of hydrocarbonoclastic bacterial communities in the oil contaminated soil sample from Egypt during bioremediation via cross-bioaugmentation.

The 42 species of hydrocarbonoclastic bacteria from the Egyptian soil (Figure 4, refer also to the Supplementary Material) belonged to the following genera: *Streptomyces* (nine species) > *Rhodococcus* (four species) > *Nocardia* (three species) > *Mycobacterium* (two species), *Mycolicibacterium* (two species). Other genera were represented with one species, each. The pristine soil (control) harbored *Nocardia neocaledoniensis* (strain 1), *S. kyeonggiensis* (strain 4), *Streptomyces scopiformis* (strain 6), and *S. bambusae* (strain 7). The mere addition of oil to this soil probably created hydrophobic conditions that resulted in the release of completely new inhabitants namely, *Arthrobacter flavus* (strain 27), *Nocardioides luteus* (strain 28), and *Streptomyces lateritius* (strain 8). Those latter taxa in turn did not show up in later phases and instead *M. vanbaalenii* (strain 15), *Z. compransoris* (strain 26), and *Streptomyces griseoflavus* (strain 9) took over the predominance after 3 months and *Z. compransoris* (strain 26), *P. benzenivorans* (strain 18), and *S. andamanensis* (strain 10) after 6 months.

The predominant bacteria in the Egyptian soil inoculated with the Kuwaiti soil were *M. vanbaalenii* (strain 15) and *B. thioparans* (strain 17). At time zero in the oily host soil, only *M. vanbaalenii* (strain 15) was detected, but *B. thioparans* (strain 17) was not and instead, *Sphingobium quisquiliarum* (strain 5), *N. neocaledoniensis* (strain 1) and *Rhodococcus globerulus* (strain 23) appeared. *M. vanbaalenii* (strain 15) remained detected after 3 months but the other inhabitants became replaced with *P. mexicana* (strain 19) and *M. vanbaalenii* (strain 15). After 6 months, the predominant taxa were *Actinotalea ferrariae* (strain 30), *X. flavus* (strain 24), and *M. vanbaalenii* (strain 31).

The bioaugmentation soil from Lebanon harbored mainly *P. benzenivorans* (strain 18) which failed to colonize the oily Egyptian soil. At time zero, this soil harbored a mixture of *M. vanbaalenii* (strain 15), *S. quisquiliarum* (strain 5), *Lentzea albidocapillata* (strain 34), *Alloactinosynnema album* (strain 35), and *R. globerulus* (strain 23). These taxa in turn became replaced with *C. segnis* (strain 37), *Streptomyces asenjonii* (strain 13), and *Z. compransoris* (strain 26) after 3 months and *A. ferrariae* (strain 30), *M. vanbaalenii* (strain 31), *Azospirillum brasilense* (strain 39) *Nocardia lijiangensis* (strain 3), and *Streptomyces chilikensis* (strain 12) after 6 months.

The bioaugmented soil from Germany harbored *X. flavus* (strain 24), *G. amicalis* (strain 25), *R. pedocola* (strain 21), *Z. compransoris* (strain 26), and *N. iowensis* (strain 2). Only *Z. compransoris* (strain 26) was detected at time zero in the oily Egyptian soil and became absolutely predominant. However, it became replaced with *Bosea thiooxidans* (strain 41), *P. mexicana* (strain 19), *S. asenjonii* (strain 13), and *X. flavus* (strain 24) after 3 months. After 6 months, another group of taxa, viz. *S. halotolerans* (strain 42), *X. flavus* (strain 24), *S. chilikensis* (strain 12), and *M. vanbaalenii* (strain 31) took over the predominance.

### Dynamics of Bacterial Communities During Bioremediation of the German Soil

**FIGURE 5:**
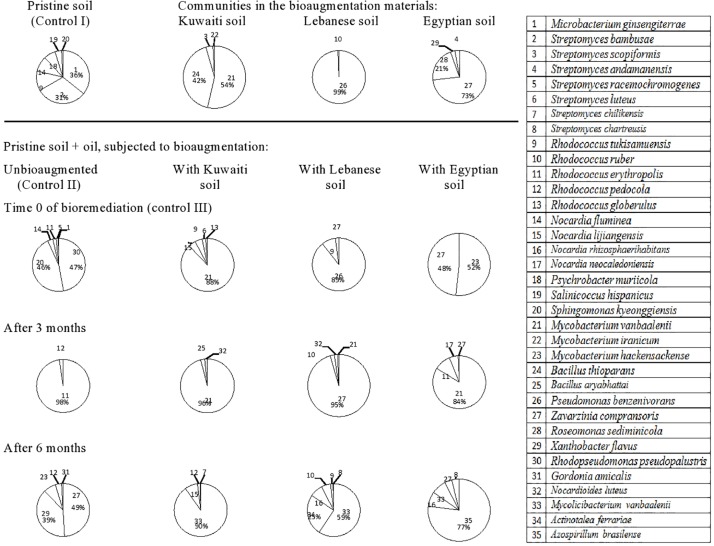
Dynamics of hydrocarbonoclastic bacterial communities in the oil contaminated soil sample from Germany during bioremediation via cross-bioaugmentation.

The 35 hydrocarbonoclastic species from the German soil (Figure 5, refer also to the Supplementary Material) belonged to the following genera: *Streptomyces* (seven species) > *Rhodococcus* (five species) > *Nocardia* (four species) > *Mycobacterium* (three species), and *Bacillus* (two species). Other genera were presented with one species, each. The pristine soil from Germany was inhabited by *Microbacterium ginsengiterrae* (strain 1), *S. bambusae* (strain 2), *Rhodococcus tukisamuensis* (strain 9), *Nocardia fluminea* (strain 14), *Psychrobacter muriicola* (strain 18), and *Salinicoccus hispanicus* (strain 19). The mere addition of oil to this soil was associated with the detection of *N. fluminea* (strain 14) and *M. ginsengiterrae* (strain 1) only, in relatively small proportions. All other inhabitants were not detected and instead, *Rhodopseudomonas pseudopalustris* (strain 30) and *S. kyeonggiensis* (strain 20) predominated together with smaller proportions of *Rhodococcus erythropolis* (strain 11) and *Streptomyces racemochromogenes* (strain 5). At later phases, all those taxa became undetectable while *R. erythropolis* (strain 11) took over the absolute predominance in the third month, and *Z. compransoris* (strain 27), *X. flavus* (strain 29), *M. hackensackense* (strain 23) and *R. pedocola* (strain 12) appeared in the sixth month.

The German soil inoculated with the Kuwaiti soil harbored *M. vanbaalenii* (strain 21) and *B. thioparans* (strain 24) as the predominant inhabitants. At time zero and after 3 months, only the former species succeeded in colonizing the host soil from Germany and persisted there in high proportions. In the sixth month, it was not detected and the predominance was taken over by *M. vanbaalenii* (strain 33).

The German soil bioaugmented with the Lebanese soil consisted predominantly of *P. benzenivorans* (strain 26), which succeeded in colonizing the host soil from Germany at time zero. This species was not detected at later phases and the soil contained *Z. compransoris* (strain 27) in the third month as the predominant inhabitant and *M. vanbaalenii* (strain 33), *A. ferrariae* (strain 34), *N. rhizosphaerihabitans* (strain 16), and *Rhodococcus ruber* (strain 10) in the sixth month.

The German soil bioaugmented with the Egyptian soil harbored as predominant species *Z. compransoris* (strain 27) and *R. sediminicola* (strain 28). The former species succeeded in colonizing the German soil at time zero, and predominated there together with *M. hackensackense* (strain 23). In the third month, the predominant species were *M. vanbaalenii* (strain 21), *R. erythropolis* (strain 11) and *N. neocaledoniensis* (strain 17) and in the sixth month, the predominant one was *A. brasilense* (strain 35) together with smaller proportions of *N. rhizosphaerihabitans* (strain 16), *M. hackensackense* (strain 23), *Z. compransoris* (strain 27), and *Streptomyces chartreusis* (strain 8).

It was noted that the bacterial communities in all the four soil samples harbored diazotrophic (N_2_-fixing) bacteria, e.g., *Gordonia*, *Rhizobium*, and *Azospirillum* in the Kuwaiti soil, *Gordonia* and *Kocuria* in the Lebanese soil, *Gordonia*, *Rhizobium*, and *Azospirillum* in the Egyptian soil and *Gordonia* and *Azospirillum* in the German soil. This is interesting from the oil-bioremediation viewpoint because nitrogen fertilizers are long known to be limiting to the microbial hydrocarbon degradation (Sorkhoh et al., 2010; Al-Bader et al., 2012). Such taxa enrich the consortia with nitrogenous compounds and thus, enhance the oil-bioremediation.

It was also noted that hydrocarbonoclastic microorganisms predominating at early stages of oil-bioremediation did not remain so at later phases. This “rule” was valid for the four soils studied. This result challenges the feasibility of bioaugmentation as an effective approach for oil-bioremediation. To recall, the bioaugmented bacterial taxa as a rule, failed to colonize the polluted, remote sites. Moreover, should they succeed in colonization, they obviously would fail in remaining predominant through the bioremediation period. In other words, the exogenous microorganisms are in most cases the weaker counter parts compared with the already existing native microorganisms. There are no similar results in the literature to compare our results with.

## Conclusion

From the systematic point of view, the bacterial communities in the four studied soils were rather similar. They consisted predominantly in all cases of higher (*Streptomyces* spp.) and lower (nocardioforms, mycobacteria) actinomycetes. In spite of this similarity, cross-bioaugmentation among the soils in most cases did not lead to successful colonization of the bioaugmented taxa, which had been even enriched in oily soils for 7 months in advance. A probable reason is that the systematically related strains are in fact basically different eco-physiologically. They are so adapted to their mother habitats that they fail to colonize and adapt to remote sites. This point should be considered carefully in bioaugmentation practices. Scanning Figures 2–5 from top downward reveals that in most cases, the oil-removal process must have been mainly due to the native (autochthonous) bacterial inhabitants of each soil. Those occurred in dynamic states depending on the ever changing parameters (nutrients, oxygen and others) in the soils during bioremediation. However, they flourished at particular phases especially in the periods with maximum oil removal rates. Examples of such autochthonous taxa are *M. vanbaalenii*, *M. vanbaalenii*, *X. flavus*, *Pseudoxanthomonas* spp., *P. benzenivorans*, *Z. compransoris* and several others. The main conclusion of this study is that bioaugmentation does not seem to be the best choice for bioremediating oil-contaminated soils. Instead, native microbial inhabitants should better do the job; their activities could be bio-enhanced by conventional managements.

## Data Availability Statement

The datasets generated for this study can be found in the GenBank, MK493198–MK493292.

## Author Contributions

SR and DA-M suggested the research topic, supervised the experimental work, and wrote the manuscript. MK did the experiments.

## Conflict of Interest

The authors declare that the research was conducted in the absence of any commercial or financial relationships that could be construed as a potential conflict of interest.
