# Corrigendum: Cross-Bioaugmentation Among Four Remote Soil Samples Contaminated With Oil Exerted Just Inconsistent Effects on Oil-Bioremediation

**DOI:** 10.3389/fmicb.2020.00211

**Published:** 2020-02-19

**Authors:** Dina M. Al-Mailem, Mayada K. Kansour, Samir S. Radwan

**Affiliations:** Microbiology Program, Department of Biological Sciences, Faculty of Science, Kuwait University, Kuwait City, Kuwait

**Keywords:** oil-bioremediation, cross-bioaugmentation, diazotrophs, hydrocarbonoclastic bacteria, remote soils

In the original article, the reference for “Di Gregorio et al., 2014” was incorrectly written as “DiGregorio, S., Castglione, M. R., Gentini, A., and Lorenzi, R. (2015). Biostimulation of the autochthonous bacterial community and bioaugmentation of selected bacterial strains for the depletion of polycyclic aromatic hydrocarbons in a historically contaminated soil. *Geophys. Res. Abstr*. 17:14690.” It should be “Di Gregorio, S., Gentini, A., Siracusa, G., Becarelli, S., Azaizeh, H., and Lorenzi, R. (2014). Phytomediated biostimulation of the autochthonous bacterial community for the acceleration of the depletion of polycyclic aromatic hydrocarbons in contaminated sediments. *Biomed. Res. Int*. 2014:891630. doi: 10.1155/2014/891630.”

The authors apologize for this error and state that this does not change the scientific conclusions of the article in any way. The original article has been updated.

