# Supplementary material for: Cross-Bioaugmentation Among Four Remote Soil Samples Contaminated With Oil Exerted Just Inconsistent Effects on Oil-Bioremediation
Source: Front Microbiol. 2019 Dec 5;10:2827. doi: 10.3389/fmicb.2019.02827 (PMC6906181; doi:10.3389/fmicb.2019.02827)
Supplement: Supplementary file 1 [file Table_1.DOCX]

Table 1. Information related to 16S rRNA gene sequencing of the microbial isolates from the four studied soil samples

| **Isolate no.** | **Total bases** | **Subdivision** | **Nearest GenBank match** | **Similarity %** | **Bases compared** | **GenBank accession no.** |
| --- | --- | --- | --- | --- | --- | --- |
| 1 | 518 | Actinobacteria | *Actinotalea ferrariae* | 100 | 518/518 | MK493198 |
| 2 | 543 | Actinobacteria | *Agromyces indicus* | 100 | 543/543 | MK493199 |
| 3 | 512 | Actinobacteria | *Alloactinosynnema album* | 99 | 516/518 | MK493200 |
| 4 | 534 | Actinobacteria | *Arthrobacter agilis* | 100 | 534/534 | MK493201 |
| 5 | 531 | Actinobacteria | *Arthrobacter echini* | 100 | 531/531 | MK493202 |
| 6 | 528 | Actinobacteria | *Arthrobacter flavus* | 100 | 528/528 | MK493203 |
| 7 | 528 | Actinobacteria | *Arthrobacter ginsengisoli* | 100 | 528/528 | MK493204 |
| 8 | 487 | Actinobacteria | *Arthrobacter nitrophenolicus* | 99 | 493/496 | MK493205 |
| 9 | 537 | Actinobacteria | *Arthrobacter phenanthrenivorans* | 100 | 537/537 | MK493206 |
| 10 | 525 | Alphaproteobacteria | *Azospirillum brasilense* | 100 | 525/525 | MK493207 |
| 11 | 534 | Bacilli | *Bacillus aryabhattai* | 100 | 534/534 | MK493208 |
| 12 | 544 | Bacilli | *Bacillus thioparans* | 100 | 544/544 | MK493209 |
| 13 | 474 | Alphaproteobacteria | *Bosea thiooxidans* | 99 | 478/480 | MK493210 |
| 14 | 454 | Alphaproteobacteria | *Caulobacter segnis* | 100 | 454/454 | MK493211 |
| 15 | 521 | Actinobacteria | *Chryseoglobus frigidaquae* | 99 | 527/530 | MK493212 |
| 16 | 462 | Alphaproteobacteria | *Ensifer alkalisoli* | 99 | 466/468 | MK493213 |
| 17 | 549 | Actinobacteria | *Gordonia amicalis* | 100 | 549/549 | MK493214 |
| 18 | 518 | Actinobacteria | *Janibacter hoylei* | 100 | 518/518 | MK493215 |
| 19 | 538 | Actinobacteria | *Kocuria polaris* | 100 | 538/538 | MK493216 |
| 20 | 497 | Actinobacteria | *Lentzea albidocapillata* | 99 | 505/509 | MK493217 |
| 21 | 530 | Actinobacteria | *Lentzea flaviverrucosa* | 100 | 530/530 | MK493218 |
| 22 | 553 | Gammaproteobacteria | *Marinobacter algicola* | 100 | 553/553 | MK493219 |
| 23 | 551 | Betaproteobacteria | *Massilia varians* | 100 | 551/551 | MK493220 |
| 24 | 535 | Actinobacteria | *Microbacterium arthrosphaerae* | 100 | 535/535 | MK493221 |
| 25 | 537 | Actinobacteria | *Microbacterium ginsengiterrae* | 100 | 537/537 | MK493222 |
| 26 | 536 | Actinobacteria | *Microbacterium pumilum* | 100 | 536/536 | MK493223 |
| 27 | 439 | Actinobacteria | *Micromonospora zamorensis* | 100 | 439/439 | MK493224 |
| 28 | 518 | Actinobacteria | *Mycobacterium arceuilense* | 99 | 520/521 | MK493225 |
| 29 | 488 | Actinobacteria | *Mycobacterium bacteremicum* | 100 | 488/488 | MK493226 |
| 30 | 535 | Actinobacteria | *Mycobacterium hackensackense* | 100 | 535/535 | MK493227 |
| 31 | 536 | Actinobacteria | *Mycobacterium iranicum* | 100 | 536/536 | MK493228 |
| 32 | 535 | Actinobacteria | *Mycobacterium vanbaalenii* | 100 | 535/535 | MK493229 |
| 33 | 521 | Actinobacteria | *Mycolicibacterium iranicum* | 100 | 521/521 | MK493230 |
| 34 | 533 | Actinobacteria | *Mycolicibacterium vanbaalenii* | 100 | 533/533 | MK493231 |
| 35 | 536 | Actinobacteria | *Nocardia fluminea* | 100 | 536/536 | MK493232 |
| 36 | 543 | Actinobacteria | *Nocardia iowensis* | 100 | 543/543 | MK493233 |
| 37 | 526 | Actinobacteria | *Nocardia lijiangensis* | 100 | 526/526 | MK493234 |
| 38 | 536 | Actinobacteria | *Nocardia neocaledoniensis* | 100 | 536/536 | MK493235 |
| 39 | 524 | Actinobacteria | *Nocardia rhizosphaerihabitans* | 100 | 524/524 | MK493236 |
| 40 | 480 | Actinobacteria | *Nocardioides luteus* | 99 | 490/495 | MK493237 |
| 41 | 567 | Bacilli | *Paenibacillus lautus* | 100 | 567/567 | MK493238 |
| 42 | 573 | Gammaproteobacteria | *Pseudomonas aeruginosa* | 100 | 573/573 | MK493239 |
| 43 | 555 | Gammaproteobacteria | *Pseudomonas benzenivorans* | 100 | 555/555 | MK493240 |
| 44 | 553 | Gammaproteobacteria | *Pseudomonas composti* | 99 | 557/559 | MK493241 |
| 45 | 556 | Gammaproteobacteria | *Pseudomonas hunanensis* | 100 | 556/556 | MK493242 |
| 46 | 524 | Gammaproteobacteria | *Pseudomonas mendocina* | 100 | 524/524 | MK493243 |
| 47 | 463 | Actinobacteria | *Pseudonocardia alni* | 100 | 463/463 | MK493244 |
| 48 | 534 | Gammaproteobacteria | *Pseudoxanthomonas japonensis* | 100 | 534/534 | MK493245 |
| 49 | 556 | Gammaproteobacteria | *Pseudoxanthomonas mexicana* | 100 | 556/556 | MK493246 |
| 50 | 437 | Gammaproteobacteria | *Psychrobacter muriicola* | 100 | 437/437 | MK493247 |
| 51 | 477 | Gammaproteobacteria | *Rheinheimera aquimaris* | 100 | 477/477 | MK493248 |
| 52 | 521 | Alphaproteobacteria | *Rhizobium alkalisoli* | 100 | 521/521 | MK493249 |
| 53 | 526 | Alphaproteobacteria | *Rhizobium petrolearium* | 100 | 526/526 | MK493250 |
| 54 | 534 | Actinobacteria | *Rhodococcus aetherivorans* | 100 | 534/534 | MK493251 |
| 55 | 536 | Actinobacteria | *Rhodococcus erythropolis* | 100 | 536/536 | MK493252 |
| 56 | 535 | Actinobacteria | *Rhodococcus globerulus* | 100 | 535/535 | MK493253 |
| 57 | 530 | Actinobacteria | *Rhodococcus jostii* | 99 | 534/536 | MK493254 |
| 58 | 535 | Actinobacteria | *Rhodococcus pedocola* | 100 | 535/535 | MK493255 |
| 59 | 535 | Actinobacteria | *Rhodococcus ruber* | 100 | 535/535 | MK493256 |
| 60 | 535 | Actinobacteria | *Rhodococcus tukisamuensis* | 100 | 535/535 | MK493257 |
| 61 | 522 | Alphaproteobacteria | *Rhodopseudomonas pseudopalustris* | 100 | 522/522 | MK493258 |
| 62 | 532 | Alphaproteobacteria | *Roseomonas aestuarii* | 100 | 532/532 | MK493259 |
| 63 | 458 | Alphaproteobacteria | *Roseomonas eburnea* | 100 | 458/458 | MK493260 |
| 64 | 467 | Alphaproteobacteria | *Roseomonas sediminicola* | 100 | 467/467 | MK493261 |
| 65 | 539 | Actinobacteria | *Saccharomonospora azurea* | 100 | 539/539 | MK493262 |
| 66 | 513 | Actinobacteria | *Saccharothrix ecbatanensis* | 100 | 513/513 | MK493263 |
| 67 | 534 | Actinobacteria | *Saccharothrix saharensis* | 100 | 534/534 | MK493264 |
| 68 | 536 | Bacilli | *Salinicoccus hispanicus* | 100 | 536/536 | MK493265 |
| 69 | 465 | Actinobacteria | *Sinomonas halotolerans* | 100 | 465/465 | MK493266 |
| 70 | 518 | Alphaproteobacteria | *Sphingobium hydrophobicum* | 100 | 518/518 | MK493267 |
| 71 | 522 | Alphaproteobacteria | *Sphingobium quisquiliarum* | 100 | 522/522 | MK493268 |
| 72 | 531 | Alphaproteobacteria | *Sphingomonas kyeonggiensis* | 100 | 531/531 | MK493269 |
| 73 | 534 | Alphaproteobacteria | *Sphingopyxis fribergensis* | 100 | 534/534 | MK493270 |
| 74 | 463 | Actinobacteria | *Streptomyces andamanensis* | 100 | 463/463 | MK493271 |
| 75 | 536 | Actinobacteria | *Streptomyces asenjonii* | 100 | 536/536 | MK493272 |
| 76 | 511 | Actinobacteria | *Streptomyces aureocirculatus* | 100 | 511/511 | MK493273 |
| 77 | 536 | Actinobacteria | *Streptomyces bambusae* | 100 | 536/536 | MK493274 |
| 78 | 539 | Actinobacteria | *Streptomyces carpaticus* | 100 | 539/539 | MK493275 |
| 79 | 531 | Actinobacteria | *Streptomyces chartreusis* | 99 | 535/537 | MK493276 |
| 80 | 528 | Actinobacteria | *Streptomyces chilikensis* | 100 | 528/528 | MK493277 |
| 81 | 434 | Actinobacteria | *Streptomyces griseoflavus* | 100 | 434/434 | MK493278 |
| 82 | 536 | Actinobacteria | *Streptomyces lateritius* | 100 | 536/536 | MK493279 |
| 83 | 506 | Actinobacteria | *Streptomyces luteus* | 99 | 520/527 | MK493280 |
| 84 | 524 | Actinobacteria | *Streptomyces neyagawaensis* | 100 | 524/524 | MK493281 |
| 85 | 535 | Actinobacteria | *Streptomyces ossamyceticus* | 100 | 535/535 | MK493282 |
| 86 | 535 | Actinobacteria | *Streptomyces pluripotens* | 100 | 535/535 | MK493283 |
| 87 | 529 | Actinobacteria | *Streptomyces polymachus* | 99 | 533/535 | MK493284 |
| 88 | 486 | Actinobacteria | *Streptomyces racemochromogenes* | 100 | 486/486 | MK493285 |
| 89 | 463 | Actinobacteria | *Streptomyces scopiformis* | 100 | 463/463 | MK493286 |
| 90 | 525 | Actinobacteria | *Streptomyces thermospinosisporus* | 100 | 525/525 | MK493287 |
| 91 | 538 | Actinobacteria | *Streptomyces wuyuanensis* | 100 | 538/538 | MK493288 |
| 92 | 527 | Alphaproteobacteria | *Xanthobacter flavus* | 100 | 527/527 | MK493289 |
| 93 | 530 | Alphaproteobacteria | *Xanthobacter polyaromaticivorans* | 100 | 530/530 | MK493290 |
| 94 | 484 | Alphaproteobacteria | *Xanthobacter tagetidis* | 99 | 494/499 | MK493291 |
| 95 | 532 | Alphaproteobacteria | *Zavarzinia compransoris* | 100 | 532/532 | MK493292 |
