# Supplementary material for: Cross-Bioaugmentation Among Four Remote Soil Samples Contaminated With Oil Exerted Just Inconsistent Effects on Oil-Bioremediation
Source: Front Microbiol. 2019 Dec 5;10:2827. doi: 10.3389/fmicb.2019.02827 (PMC6906181; doi:10.3389/fmicb.2019.02827)
Supplement: Supplementary file 2 [file Table_2.DOCX]

**Molecular analysis of the microbial isolates from the four studied soil samples**

For characterization of the isolates, their 16S rRNA-genes were sequenced and the sequences compared with those of type strains in GenBank data base. To extract the total genomic DNA, 300 mg of the fresh 36-hour bacterial biomass was homogenized in 100 µl of PrepMan Ultra Sample Preparation Reagent (Applied Biosystems, USA) and 200 µl molecular water (Sigma, UK). The mixture was incubated in a water bath for 10 min at 100 ^o^C, cooled for 2 min and then centrifuged at 14,000 x *g* for 3 min to collect the DNA-containing supernatant. The 16S rRNA-genes were amplified by the polymerase chain reaction (PCR). The reaction mixture contained puReTaq Ready-To-Go PCR Beads (Amersham Biosciences, UK), 1 µl (25 ng) of DNA template, and 1 µl each of the universal primer combinations GM5F (50-CCTACGGGAGGCAGCAG-30) and 907R (50-CCGTCAATTCMTTTGAGTTT-30) (Santegoeds, et al., 1998). The reaction volume was made up to 25 µl with molecular water. Amplification was done in a Veriti Thermal Cycler (Applied Biosystems, USA) by touch-down PCR in which the initial denaturation was at 95 ^o^C for 5 min, and the annealing temperature started at 65 ^o^C and decreased by 1 ^o^C every cycle to 55 ^o^C; 15 additional cycles were carried out at this temperature. The PCR products were purified using a QIA quick PCR purification kit (Qiagen, USA) to remove the Taq polymerase, primers and dNTPs. Partial sequencing of the 16S rRNA-gene was done using a BigDye version Terminator Kit (Applied Biosystems, USA); 20 ng of the DNA template was added to 2 µl of a Big Dye v 3.1 terminator and 2 µl of Big Dye Terminator v 1.1, v 3.1 5X sequencing buffer; l µl of either 907R or GM5F was added to the mixture, and the final volume was brought up to 10 µl with molecular water. Labelling was completed in a Veriti Thermal Cycler (Applied Biosystems, USA) using one cycle of 96 ^o^C for l min, then 25 cycles of l min at 96 ^o^C, 5 s at 50 ^o^C and 4 min at 60 ^o^C. The pure template DNA samples were processed in a 3130xl genetic analyzer (Applied Biosystems, USA). Sequencing analysis version 5.2 software (Applied Biosystems, USA) was used to analyze the results. Sequences were subjected to basic local alignment search tool analysis with the National Center for Biotechnology Information (NCBI; Bethesda, MD, USA) GenBank database (Altschul, et al., 1997).
